# Editorial: The global syndemic of obesity and type 2 diabetes in childhood

**DOI:** 10.3389/fped.2024.1445172

**Published:** 2024-07-10

**Authors:** Anna Di Sessa

**Affiliations:** Department of Woman, Child, and General and Specialized Surgery, University of Campania “Luigi Vanvitelli”, Naples, Italy

**Keywords:** obesity, type 2 diabetes, children, syndemic, cardiometabolic risk, treatment

**Editorial on the Research Topic** The global syndemic of obesity and type 2 diabetes in childhood

In the face of alarming epidemiological data of epidemic proportions worldwide, obesity and Type 2 diabetes (T2D) have become a dramatic syndemic in childhood. Indeed, the increasing burden of long-term cardiovascular and metabolic consequences of this syndemic—mainly mediated by insulin resistance (IR)- complicates the already difficult management of these patients and represents a major challenge for researchers investigating the intricate field of childhood obesity.

In addition to the need for a deeper understanding of the pathophysiology, prevention and treatment strategies are of paramount importance to counteract the intrinsically higher cardiometabolic risk of children with obesity and T2D. In particular, lifestyle interventions remain the cornerstone of treatment for these young patients.

Huang et al. explored the association between T2D-related risks and sociodemographic status in Chinese patients by analyzing data from the Global Burden of Disease Study 2019. In addition, secondary analyses of year, age, sex, summary exposure value (SEV), and sociodemographic index (SDI) were conducted.

Notably, the number of deaths and disability-adjusted life years (DALYs) associated with T2D has nearly tripled over the past two decades, with a greater burden of disease in male patients.

Despite the alarming epidemiological datai, an inverse association between T2D-related burden and SDI was demonstrated. Among the determinants, dietary risks, air pollution, tobacco, and high body mass index (HBMI) were identified as the major risk factors. The latter in particular was found to be a key player in T2D burden, underscoring the role of modifiable lifestyle factors in this context.

To date, both prevention and treatment strategies are mainly based on physical activity, but their characterization is still less defined.

The challenging question of the optimal balance of time spent in daily physical activity behaviors (the so-called Goldilocks Day) in children with obesity was examined in an interesting study by Rasmussen et al. A comprehensive evaluation including adiposity index assessment and accelerometer-measured 24-h physical activity behaviors was conducted in a cohort of 659 Czech children and adolescents (aged 8–18-years). Certain adiposity parameters such as visceral adipose tissue and fat mass percentage were found to be significantly associated with physical activity behaviors. A duration of 8.5 h of sleep, 10.8 h of sedentary behavior (SB), 3.9 h of light physical activity (LPA), and 0.8 h of moderate-to-vigorous physical activity (MVPA) resulted in the Goldilocks Day in children. Similarly, it consisted of 7.5 h of sleep, 12.4 h of SB, 3.6 h of LPA, and 0.5 h of MVPA in adolescents.

Given the pandemic proportions of childhood obesity and its cardiometabolic burden, there is increasing evidence to shed light on the complex pathophysiology of the disease. For this reason, the gut microbiota has recently attracted scientific interest. Li et al. analyzed data from the Microbiome Genome Consortium for gut microbiota (GM) and the Early Growth Genetics Consortium for childhood obesity (CO) and childhood body mass index (CBMI), and demonstrated an association of 8 bacterial genera with pediatric obesity risk. In particular, 3 childhood obesity-related GMs including the genera Akkermansia, Intestinibacter, and Butyricimonas were identified after validation of the results in the replication dataset. Of note, a significant negative association of Akkermansia with the risk of both CO and CBMI was reported.

In this intricate context, the gut metagenome has also emerged as an interesting but still poorly understood subject of study. Carrizales-Sánchez et al. investigated the taxonomic composition of the gut microbiome and its potential association with metabolic dysregulation and proinflammatory effects in a cohort of young patients with T2D and metabolic syndrome (MetS) compared to healthy children. Interestingly, gut microbial dysbiosis characterized by an increase in facultative anaerobes and a decrease in strict anaerobes was found in children with T2D and MetS compared to healthy subjects. Notably, these metabolic imbalances may not only affect intermediate host metabolism but also specific molecular patterns favoring proinflammatory activity. Furthermore, specific viruses were found to be significantly associated with proinflammatory cytokines involved in T2D and MetS. Overall, these changes may lead to a progression of the major risk factors of both diseases (e.g., IR, dyslipidemia, and adiposity parameters), providing insightful perspectives in this intriguing field of research.

Given the relevant multifaceted impact of childhood obesity on cardiometabolic health, findings from studies included in this Research Topic may provide the basis for further advances in the complex management of these young patients, as suggested by the intriguing role of the gut microbiota as a potential therapeutic target.

Remarkably, these findings may not only contribute to the development of novel prevention and intervention strategies but also to a deeper understanding of the pathophysiology of childhood obesity, shedding new light on this complex area of research.

